# Zero-augmented beta-prime model for multilevel semi-continuous data: a Bayesian inference

**DOI:** 10.1186/s12874-022-01736-0

**Published:** 2022-11-02

**Authors:** Naser Kamyari, Ali Reza Soltanian, Hossein Mahjub, Abbas Moghimbeigi, Maryam Seyedtabib

**Affiliations:** 1 Department of Biostatistics and Epidemiology, School of Health, Abadan University of Medical Sciences, Abadan, Iran; 2 Modeling of Noncommunicable Diseases Research Center, Hamadan University of Medical Sciences, Street of Mahdieh, Hamadan, Iran; 3 Research Center for Health Sciences, School of Public Health, Hamadan University of Medical Sciences, Hamadan, Iran; 4 Department of Biostatistics and Epidemiology, School of Health, Research Center for Health, Safety and Environment, Alborz University of Medical Sciences, Karaj, Iran; 5 Department of Biostatistics and Epidemiology, School of Health, Ahvaz Jundishapur University of Medical Sciences, Ahvaz, Iran

**Keywords:** Bayesian framework, Non-negative data, Two-part mixed-effects model, Skew distributions, Pharmaceutical expenditure

## Abstract

Semi-continuous data characterized by an excessive proportion of zeros and right-skewed continuous positive values appear frequently in medical research. One example would be the pharmaceutical expenditure (PE) data for which a substantial proportion of subjects investigated may report zero. Two-part mixed-effects models have been developed to analyse clustered measures of semi-continuous data from multilevel studies. In this study, we propose a new flexible two-part mixed-effects model with skew distributions for nested semi-continuous cost data under the framework of a Bayesian approach. The proposed model specification consists of two mixed-effects models linked by the correlated random effects: Part I) a model on the occurrence of positive values using a generalized logistic mixed model; and Part II) a model on the magnitude of positive values using a linear mixed model where the model errors follow skew distributions including beta-prime (BP). The proposed method is illustrated with pharmaceutical expenditure data from a multilevel observational study and the analytic results are reported by comparing potential models under different skew distributions. Simulation studies are conducted to assess the performance of the proposed model. The DIC_3_, LPML, WAIC, and LOO as the Bayesian model selection criteria and measures of divergence used to compare the models.

**Supplementary Information:**

The online version contains supplementary material available at 10.1186/s12874-022-01736-0.

## Introduction

Semi-continuous data featured with an excessive proportion of zeros and right-skewed positive values arise frequently in health economics and health services research [1]. Examples include alcohol consumption, household-level consumption of food items, medical cost, and substance abuse symptom scales. Statistical models with normality assumptions ignoring the skewness and the spike at zero are not suitable for this type of data and may lead to substantial bias and incorrect statistical inferences. Two-part (zero-augmented) models, originating in econometrics [2, 3], have been developed extensively in the last three decades to analyse this type of data and have been applied to scientific fields other than economics such as clinical research and health services. In two-part models, we view a semi-continuous variable as the result of two processes: one binomial process determining whether the positive value occurs and one continuous process determining the actual value given it is nonzero. Therefore, a two-part model consists of two components, with the first component (i.e., Part I) modelling the probability of a response being positive using the probit or logistic regression, and the second component (i.e., Part II) modelling the conditional mean of the positive values (given positive values occurred) using the continuous regression. According to the data structures, various methods have been developed for analysing cross-sectional and longitudinal semi-continuous data [3–7]. Olsen and Schafter [6] first extended the two-part models developed by Duan et al. [3] and Manning et al. [8] for cross-sectional data to the longitudinal setting by introducing correlated random-effects into the logit and log-normal components, respectively, and applied them to longitudinal alcohol data. In the two-part mixed-effects models, the binomial process is typically modelled with mixed-effects logistic or probit regression, and the continuous process is naturally modelled via linear mixed models (LMMs). The random-effects in the two components are generally assumed to be correlated through a multivariate normal distribution structure. Ignoring the between-component association mistakenly can yield biased estimates in the second part of the model [9]. The correlated random-effects can capture not only the between-component association but also the within-subject correlation among repeated measurements collected from the same individual and nested data. A between-component correlation means that the process giving rise to the positive values is related to the magnitude of the observed value given that a positive response occurred. For example, in a data collection of self-reported daily drinks (DDD) where zero represents no daily drinks and the continuous positive values reflect the mean of drinks per day, a positive correlation suggests that an individual with high odds of drinking tended to drink more alcohol [10].

For the positive part of a semi-continuous variable, LMMs with a normality assumption were used by Husted et al. [11] and Su et al. [9]. However, the positive part of a semi-continuous variable is often right-skewed. The logarithmic transformation was the most commonly used approach to correct the skewness [6, 7] and other monotone increasing functions such as Box-Cox transformation that would make the positive component approximately normal were also explored [12, 13]. The limitations with data transformation in Part II include reduced information, difficulty in interpreting the results and possible heteroscedasticity [13, 14]. An alternative approach is to use generalized linear mixed models (GLMMs) with distributions in the exponential family that can model skewed data, such as Log-Normal, Log-Skew-Normal [15], Gamma [13], Inverse Gamma, Inverse Gaussian [16], Beta [17], Bridge [18], Generalized Gamma family, and Weibull distributions [19]. It is noted that GLMMs often involve complicated iterative procedures in estimation which may lead to intensive computation burden and non-convergence issues. It would be most effective to use a flexible distribution to model the right skewed positive values in two-part models. Recently, studies have been presented using the beta-prime (BP) distribution to fit long-tail semi-continuous responses [10, 20, 21]. There is very limited research on the application of this skew distribution in a two-part mixed-effects model [22]. This study is an extension of Kamyari et al. (2021), where random effects are added to the linear predictor terms by using real two-level data.

Parameter estimations in two-part modelling could be computationally difficult. For two-part models with independent random-effects, maximum likelihood estimates (MLE) can be derived by fitting separate mixed-effects model to each part [1]. For the correlated two-part mixed-effects model with log-normal distribution on the positive values, Olsen and Shafter [6] and Tooze et al. [7] developed different maximum likelihood approaches. Several authors have proposed Bayesian approaches to fit the two-part models [17, 23–27]. For example, Cooper et al. [23] used a Bayesian approach via Markov Chain Monte Carlo (MCMC) to fit a probit-lognormal correlated two-part model on medical cost data.

As a result, In this study, we propose a two-part mixed-effects model with a logistic mixed model on the occurrence of positive values and a GLMM with BP distribution on the continuous positive values using the Bayesian approach via MCMC procedure with application to a three-level pharmaceutical expenditure data. The data used for this study was extracted from the Iranian pharmaceutical expenditure (PE-2018) survey. The survey was a cross-sectional study that had been conducted by the National Center for Health Insurance Research, Iran Health Insurance Organization. PE-2018 is a dataset of yearly pharmaceutical expenditure per person conducted in 429 cities in Iran.

The rest of the article is organized as follows. At the beginning of Methods, we describe the BP regression model. In Model specification, we present the two-part mixed effects model for responses with BP distribution. In Numerical study, we apply the proposed methodologies to real data and report the analysis results. In Simulations, simulation studies are conducted to assess the performance of the proposed models. Finally, we conclude the article with a discussion in Discussion.

## Methods

### Beta-prime regression model

The BP distribution [28, 29] is also known as inverted beta distribution or beta distribution of the second kind, often the model of choice for fitting semi-continuous data where the response variable is measured continuously on the positive real line (*Y* > 0) because of the flexibility it provides in terms of the variety of shapes it can accommodate. The probability density function (PDF) of a BP distributed random variable *Y* parameterized in terms of its mean *μ* and a precision parameter *ψ* is given by

1 $$f\left(y|\mu, \psi \right)=\frac{y^{\mu \left(\psi +1\right)-1}\ {\left(1+y\right)}^{-\left[\mu \left(\psi +1\right)+\psi +2\right]}}{B\left(\mu \left(1+\psi \right),\psi +2\right)}\kern1em ,\kern0.75em y>0$$

where *B* denote the beta function, *μ* > 0, *ψ* > 0, *E*(*Y*) = *μ*, and *Var*(*Y*) = (*μ*(1 + *μ*))/*ψ*.

**Fig. 1:**
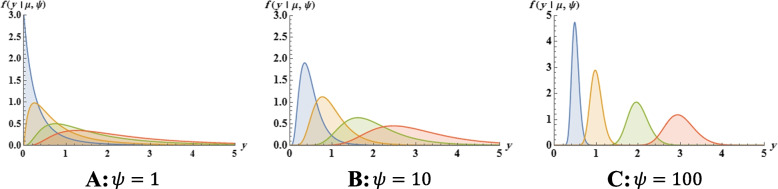
Plots of probability density function of the BP distribution considering the following values of *μ* = 0.5 (blue), *μ* = 1.0 (yellow), *μ* = 2.0 (green) and *μ* = 3.0 (red). Graphs plotted by using Wolfram Mathematica

Figure 1 displays some plots of the density function in Eq. (1) for some parameter values. It is evident that the distribution is very flexible and it can be an interesting alternative to other distributions with positive support. Figure 1 shows that for a fixed value of the mean *μ*, higher values of *ψ* lead to a reduction of *Var*(*Y*), and vice versa. If *Y* has PDF as in Eq. (1), we denote *Y*~*BP*(*μ*, *ψ*). Next, to connect the covariate vector ***X***_***k***_, *k* = 1, …, *m* to the random sample *Y*_1_, *Y*_2_, …, *Y*_*m*_ of *Y*, we use a suitable link function *g*_1_ that maps the mean interval (0, +∞) onto the real line. This is given as ${g}_1\left({\mu}_k\right)={\boldsymbol{X}}_{\boldsymbol{k}}^{\prime}\boldsymbol{\beta}$, where ***β*** is the vector of regression parameters, and the first element of ***X***_***k***_ is 1 to accommodate the intercept. The precision parameter *ψ*_*k*_ is either assumed constant [30, 31] or regressed onto the covariates [30, 32] via another link function *g*_2_, such that ${g}_2\left({\psi}_k\right)={\boldsymbol{Z}}_{\boldsymbol{k}}^{\prime}\boldsymbol{\gamma}$, where ***Z***_***k***_ is a covariate vector (not necessarily similar to ***X***_***k***_) and *γ* is the corresponding vector of regression parameters. Similar to ***X***_***k***_, ***Z***_***k***_ also accommodates an intercept. The link functions *g*_1_ : *ℝ*^*y* > 0^ ⟶ *ℝ* and *g*_2_ : *ℝ*^*y* > 0^ ⟶ *ℝ* must be strictly monotone, positive and at least twice differentiable, such that ${\mu}_k={g}_1^{-1}\left({\boldsymbol{X}}_{\boldsymbol{k}}^{\prime}\boldsymbol{\beta} \right)$ and ${\psi}_k={g}_2^{-1}\left({\boldsymbol{Z}}_{\boldsymbol{k}}^{\prime}\boldsymbol{\gamma} \right)$, with ${g}_1^{-1}(.)$ and ${g}_2^{-1}(.)$ being the inverse functions of *g*_1_(.) and *g*_2_(.), respectively. We can estimate the parameters of the BP regression model defined in Eq. (1) using the gamlss function in the R (≥ 3.3.0) language [33] with a package of the same name [34].

### Model specification

In this section, we present our model for the yearly pharmaceutical expenditure record in three levels. However, our model can be adapted easily to more complicated settings.

In the three-level pharmaceutical expenditure record data, level 3 is the province; level 2 is the city level that nested within province; and level 1 is the subject level that is nested in cities. There are two types of correlations at different levels in the pharmaceutical expenditure data. The first type exists at the province level, where cost records of the same province are correlated. For each subject, there also exists another correlation within each city. Thus, a three-level random effects two-part model is more appropriate for the analysis of the pharmaceutical expenditure data. We are interested in modelling a three-level semi-continuous pharmaceutical expenditure data, characterized by a large portion of zeros and continuous positive values. We define notations as follows. Suppose that we observe cost record *y*_*ijk*_ for the *k-*th subject of city *j* within province *i*, where *i* = 1, 2, ..., n, *j* = 1, 2, ..., *n*_*i*_, and *k* = 1, 2, ..., *m*_*ij*_. The total number of cities is $J=\sum_{i=1}^n{n}_i$, and the total number of subjects is $N=\sum_{i=1}^n\sum_{j=1}^{n_i}{m}_{ij}$. Let *ω*_*ijk*_ = *I*(*y*_*ijk*_ > 0) denote the indicator of *y*_*ijk*_ being nonzero. Define by ***X***_***ijk***_ the covariate vectors for the fixed effect. Let *p*_1*i*_ and *p*_2*i*_ be the correlated random effects in the province level with joint density (*p*_1*i*_, *p*_2*i*_) for parts I and II of our proposed model, respectively. Similarly, define *c*_1*ij*_ and *c*_2*ij*_ to be the correlated random effects with joint density (*c*_1*ij*_, *c*_2*ij*_) in the city level. In this paper, we assume that

2 $$\left({p}_{1i},{p}_{2i}\right)\sim N\left(o,{\boldsymbol{\Sigma}}_{1}=\left(\begin{array}{cc}{\sigma}_{p_1}^2& {\rho}_p{\sigma}_{p_1}{\sigma}_{p_2}\\ {}{\rho}_p{\sigma}_{p_1}{\sigma}_{p_2}& {\sigma}_{p_2}^2\end{array}\right)\right)\ \textrm{and}\ \left({c}_{\text{1ij}},{c}_{\text{2ij}}\right)\sim N\left(o,{\boldsymbol{\Sigma}}_2=\left(\begin{array}{cc}{\sigma}_{c_1}^2& {\rho}_c{\sigma}_{c_1}{\sigma}_{c_2}\\ {}{\rho}_{c}{\sigma}_{c_2}{\sigma}_{c_1}& {\sigma}_{c_2}^2\end{array}\right)\right)$$

with **Σ**_1_ and **Σ**_2_ being positive definite matrices. We also assume that (*p*_1*i*_, *p*_2*i*_) and (*c*_1*ij*_, *c*_2*ij*_) are independent for all *i’s* and *j’s*. Denote by *e*_*ijk*_ the error term for the positive value of *Y*_*ijk*_. We assume that ${e}_{ijk}\sim N\left(o,{\sigma}_e^2\right)$ is independent of random effects *p*_1*i*_, *p*_2*i*_, *c*_1*ij*_, and *c*_2*ij*_. Define *τ*_*ijk*_ = *P*(*ω*_*ijk*_ = 1| *p*_1*i*_, *c*_1*ij*_) to be the probability of non-zero value for *Y*_*ijk*_.

To obtain interpretable covariate effects on the marginal mean, we propose the following marginalized two-part model that parameterizes the covariate effects directly in terms of the marginal mean, *μ*_*ijk*_ = *E*(*Y*_*ijk*_), on the original (i.e., untransformed) data scale. The marginalized two-part model with random (cluster) effects for the zero and the continuous components, respectively, specifies the linear predictors.

Part I:

3 $${\displaystyle \begin{array}{c}{\omega}_{ijk}\sim Bernoulli\left({\tau}_{ijk}\right)\\ {} logit\left({\tau}_{ijk}\right)= logit\left({\Pr}\left({Y}_{ijk}\ne 0|{p}_{1i},{c}_{1 ij}\right)\right)={\boldsymbol{X}}_{1 ij k}^{\prime}\times \boldsymbol{\alpha} +{p}_{1i}+{c}_{1 ij}\\ {}=\left({\alpha}_{0}+{p}_{1i}+{c}_{1 ij}\right)+\sum_{\gamma =1}^r{\alpha}_{\gamma }{x}_{\gamma_i}\end{array}}$$

Part II:

4 $${\displaystyle \begin{array}{c}\left({Y}_{ijk}|{Y}_{ijk}>0\right)\sim Beta\ Prime\left({\upmu}_{\textrm{ijk}},\psi \right)\\ {}{\upmu}_{\textrm{ijk}}=E\left({Y}_{ijk}>0|{p}_{2i},{c}_{2 ij}\right)={\exp}\left({\boldsymbol{X}}_{2 ij k}^{\prime}\times \boldsymbol{\beta} +{p}_{2i}+{c}_{2 ij}+{e}_{ijk}\right)\\ {}={\exp}\left(\left({\beta}_0+{p}_{2i}+{c}_{2 ij}\right)+\sum_{\theta =1}^q{\beta}_{\theta }{x}_{\theta_i}+{e}_{ijk}\right)\end{array}}$$

where ***X***_1*N* × (*r* + 1)_ and ***X***_2*N* × (*q* + 1)_ have full rank *r* and *q* for the zero and the continuous components, respectively; ***α***_(*r* + 1) × 1_ and ***β***_(*q* + 1) × 1_ are the corresponding vectors of regression coefficients. As seen in relations (3) and (4), the mixing probability and mean of the component of the continuous parts are linked to the independent variables through logit and logarithmic link functions. The vectors ***p***_1_ = (*a*_11_, *a*_12_, …, *a*_1*m*_)^′^ and ***p***_2_ = (*a*_21_, *a*_22_, …, *a*_2*m*_)^′^ denote random effects of the third level in the components of logistic and continuous, respectively, whereas ${\boldsymbol{c}}_1={\left({b}_{111},\dots, {b}_{11{n}_1},\dots, {b}_{1m1},\dots, {b}_{1m{n}_m}\right)}^{\prime }$ and ${\boldsymbol{c}}_2={\left({b}_{211},\dots, {b}_{21{n}_1},\dots, {b}_{2m1},\dots, {b}_{2m{n}_m}\right)}^{\prime }$ are the random effects of the second level. For simplicity of interpretation and mathematical calculations, the random effects (*p*_1_, *p*_2_) and (*c*_1_, *c*_2_) are assumed to be independent and normally distributed with mean zero and variances ${\sigma}_{p_1}^2,{\sigma}_{p_2}^2,{\sigma}_{c_1}^2$ and ${\sigma}_{c_2}^2$, respectively [35, 36]. The error terms ${e}_{ijk}\sim N\left(0,{\sigma}_e^2\right)$ are also assumed to be normal distribution and independent of the random effects at both levels 2 and 3.

Again and according to the data structure in this study (three-level data), ${\boldsymbol{X}}_{1 ijk}^{\prime }$ is the vector of covariates for the *k-*th measurement at the *j-*th city (level-2) at the *i-*th province (level-3) for the binary part and ${\boldsymbol{X}}_{2 ijk}^{\prime }$ is the vector of covariates for the *k-*th measurement at the *j-*th city (level-2) at the *i-*th province (level-3) for the continuous part. The two parts might have common covariates or completely different ones. ***α*** is the vector of model coefficients corresponding to the binary part and ***β*** is the vector of coefficients corresponding to the continuous part conditional on the values being non-zero. The model can be easily extended to include higher-level random effects.

The conditional PDF for *y*_*ijk*_ is expressed as:

$${\displaystyle \begin{array}{c}\textrm{f}\left({\textrm{y}}_{\textrm{ijk}}|{\textrm{p}}_{\textrm{1i}},{\textrm{p}}_{\textrm{2i}},{\textrm{c}}_{\textrm{1ij}},{\textrm{c}}_{\textrm{2i j}}\right)={\left[1-{\uptau}_{\textrm{ijk}}\right]}^{1-{\upomega}_{\textrm{ijk}}}\times {\left[{\uptau}_{\textrm{ijk}}\times \textrm{BP}\left({\textrm{y}}_{\textrm{ijk}}|{\textrm{p}}_{\textrm{2i}},{\textrm{c}}_{\textrm{2ij}}\right)\right]}^{\upomega_{\textrm{ijk}}}\\ {}=\left\{{\left[1-{\uptau}_{\textrm{ijk}}\right]}^{1-{\upomega}_{\textrm{ijk}}}{\uptau_{\textrm{ijk}}}^{\upomega_{\textrm{ijk}}}\right\}\times {\left[\textrm{BP}\left({\textrm{y}}_{\textrm{ijk}}|{\textrm{p}}_{\textrm{2i}},{\textrm{c}}_{\textrm{2ij}}\right)\right]}^{\upomega_{\textrm{ijk}}}\end{array}}$$

Generally, the estimation of parameters ***α***, ***β***, *ψ*, **Σ**_**1**_ and **Σ**_**2**_ is based on the likelihood function of data given as:

5 $${L}_i=\int \prod\nolimits_{j=1}^{n_i}\left[\int \exp \left({l}_{ij}^1\right)\exp \left({l}_{ij}^2\right)\phi \left({c}_{1 ij},{c}_{2 ij}\right)d{c}_{1 ij}\ d{c}_{2 ij}\kern0.5em \right]\phi \left({p}_{1i},{p}_{2i}\right)\ d{p}_{1i}\ d{p}_{2i}$$

where the log-likelihood for the binary part is

6 $${\displaystyle \begin{array}{c}{l}_{ij}^1={\sum}_{k=1}^{m_{ij}}\left[{\omega}_{ij k}\log \left({\tau}_{ij k}\right)+\left(1-{\omega}_{ij k}\right)\log \left(1-{\tau}_{ij k}\right)\right]\\ {}=\sum_{k=1}^{m_{ij}}\left[{\omega}_{ij k}\ \textrm{logit}\left({\tau}_{ij k}\right)+\log \left(1-{\tau}_{ij k}\right)\right]\end{array}}$$

and the log-likelihood for the continuous part is

7 $$l_{ij}^2=-\frac1{2\sigma_e^2}{\textstyle\sum_{k=1}^{m_{ij}}}\omega_{ijk}\;e_{ijk}^2-{\textstyle\sum_{k=1}^{m_{ij}}}\omega_{ijk}\log\sigma_e+constant$$

$$\textrm{With}\ {e}_{ijk}={\boldsymbol{Y}}_{ijk}-{\boldsymbol{X}}_{2 ij k}^{\prime}\boldsymbol{\beta} -{\boldsymbol{p}}_{2i}-{\boldsymbol{c}}_{2 ij}$$

In this likelihood function (Eq. 5), *ϕ*(*p*_1*i*_, *p*_2*i*_) and *ϕ*(*c*_1*ij*_, *c*_2*ij*_) represents the bivariate normal distribution for the random effects with mean vector of zeros and variance–covariance matrix **Σ**_1_ and **Σ**_2_ for zero and non-zero part respectively. As can be seen from Eq. (5), the likelihood function involves the integral with respect to the multivariate normal PDF. Parameter estimation in the proposed models can be computationally difficult as the likelihood function depends on analytically intractable integrals of a non-linear function with respect to the multivariate normal distribution of random-effects.

### Bayesian inferential framework

The parameters in part I and II were individually estimated within a Bayesian inferential framework with MCMC sampling of the posteriors.

Let Θ = (***α***, ***β***, *ψ*, **Σ**_**1**_, **Σ**_**2**_) be the collection of unknown population parameters in models (2), (3) and (4). To complete the Bayesian formulation, we specify mutually independent prior distributions for all the unknown parameters as follows:

8 $${\displaystyle \begin{array}{c}\upalpha \sim {\textrm{N}}_{\textrm{r}}\left({\upalpha}_0,{\Lambda}_1\right),\upbeta \sim {\textrm{N}}_{\textrm{q}}\left({\upbeta}_0,{\Lambda}_2\right)\\ {}\uppsi \sim \textrm{IG}\left(\upalpha, \upbeta \right),{\Sigma}_1^{-1}\sim \textrm{IW}\left({\Omega}_1,{\upnu}_1\right),{\Sigma}_2^{-1}\sim \textrm{IW}\left({\Omega}_2,{\upnu}_2\right)\end{array}}$$

where by considering the information available from literature [1, 16, 37] and range of the parameters Normal (N), Inverse Gamma (IG), and Inverse Wishart (IW) distributions are chosen to simplify computations.

Let the observed data $\mathfrak{D}=\left\{\left({\omega}_{ij},{y}_{ij},{x}_{1 ij},{x}_{2 ij}\right);i=1,\dots, n;j=1,\dots, {n}_i\right\}$, *f*(.) be a density function, *f*(.| .) be a conditional density function and *h*(.) be a prior density function. We assume that the parameters in Θ are independent of each other; that is:

$$h\left(\Theta \right)=h\left(\upalpha \right)h\left(\upbeta \right)h\left(\psi \right)h\left({\Sigma}_1\right)h\left({\Sigma}_2\right).$$

After specifying the models for the observed data and prior distributions of the unknown model parameters, we can draw samples for the parameters based on their posterior distributions under the Bayesian framework. Therefore, the joint posterior density of Θ, conditional on $\mathfrak{D}$, can be determined by

9 $$f\left(\Theta |\mathfrak{D}\right)\propto \left\{\prod\nolimits_{i=1}^n\int \prod\nolimits_{j=1}^{n_i}\left[\int \exp \left({l}_{ij}^1\right)\exp \left({l}_{ij}^2\right)\phi \left({c}_{1 ij},{c}_{2 ij}\right)d{c}_{1 ij}\ d{c}_{2 ij}\kern0.5em \right]\phi \left({p}_{1i},{p}_{2i}\right)\ d{p}_{1i}\ d{p}_{2i}\right\}h\left(\Theta \right)$$

The integral in (9) has a high dimension and does not have a closed solution. Analytic approximations to the integrals may not be accurate enough. So, the direct calculation of the posterior distribution of Θ based on the observed data $\mathfrak{D}$ is prohibitive [1]. As an alternative, posterior computation of Θ can proceed using a MCMC procedure via Gibbs sampling or Metropolis-Hastings (M-H) algorithm. While the Gibbs sampler relies on conditional distributions [23, 38–40] the Metropolis-Hastings sampler uses a full joint density distribution to generate a candidate draws [38, 41]. Certainly, there is a large body of work on other computational approaches to sampling (slice sampling, adaptive rejection sampling, Hamiltonian Monte Carlo, etc.); covering such methods is beyond the scope of this study. In an initial review of the software, we concluded that it would be faster to use the OpenBUGS software. Moreover, due to the high volume of data (calculations) and the time limitation, we could not check the performance of other software. However, OpenBUGS was chosen because of its generality and simplicity. The associated OpenBUGS code is available in Additional file 1: Appendix A.1.

### Model complexity and fit

There are a variety of methods to select the model that best fits the data. However, in this research article, we focus on the log pseudo marginal likelihood (LPML) and a modified observed deviance information criterion, denoted here by DIC_3_. In addition, we use of two emerging model selection methods, namely leave-one-out cross-validation (LOO-CV) and widely available information criterion (WAIC), due to their fully Bayesian nature.

The Bayesian Deviance Information Criterion (DIC_3_) [42] is used to compare the models fitted. It is defined by

$${\textrm{DIC}}_3=\overline{D}\left(\theta \right)+{p}_D$$

where $\overline{D}\left(\theta \right)=-2E\left\{\log \left[p\left(y|\uptheta \right)\right]|y\right\}$ is the posterior mean deviance taken as Bayesian measure of fit, $p\left(y|\uptheta \right)=\prod_{i=1}^np\left({y}_i|\uptheta \right)$, *E*{log[*p*(*y*| θ)]| *y*} is the posterior expectation of log[*p*(*y*| *θ*)] and *p*_*D*_ is the effective number of parameters representing model complexity. The DIC_3_ is a natural generalization of the Akaike Information Criterion (AIC) [43] and interpreted as a Bayesian measure of fit penalized for increased model complexity. The DIC_3_ was developed to solve the problem of determining the ‘effective’ number of parameters (*p*_*D*_) in complex non-nested hierarchical models and its computation has been coded into the latest version of WinBUGS (1.4). As for the usual DIC, minimum DIC_3_ estimates the model that will make the best short-term predictions [42]. Note that the DIC_3_ is only comparable across models with exactly the same observed data.

The LPML [44] is another measure for comparing models that derived from the conditional predictive ordinate (CPO) statistics and is one of the most widely used model selection criteria available in WinBUGS. It is derived from the posterior predictive distribution. For our proposed models, a closed form of the *CPO*_*i*_ is not available. However, a Monte Calro estimate of *CPO*_*i*_ can be obtained by using a single MCMC sample from the posterior distribution of θ, through a harmonic-mean approximation proposed by [45], as $\hat{CPO_i}={\left\{\frac{1}{K}\sum_{i=1}^Kg{\left({y}_i|{\uptheta}^{(i)}\right)}^{-1}\right\}}^{-1}$, where θ^(1)^, …, θ^(*K*)^ is a post burn-in sample of size *K* from the posterior distribution from θ, and *g* is the marginal distribution of *Y* (integrated over the random effects). A summary statistic of the *CPO*_*i*_ is the LPML, defined by $LPML=\sum_{i=1}^n\log \left(\hat{CPO_i}\right)$. Larger values of LPML indicate better fit.

The Watanabe-Akaike (or widely applicable) information criterion (WAIC) [46, 47] is closely related to the more widely known DIC measure, which is based on a deviance. The WAIC is a more fully Bayesian approach for estimating out-of-sample expectation. In general, the WAIC is defined as:

$$WAIC=2{p}_{WAIC}-2 LPPD$$

The deviance term in DIC is ${\log}\left(p\left(y|\overset{\sim }{\theta}\right)\right)$ where $\overset{\sim }{\theta }$ is a point estimate of θ. For WAIC, this term is replaced by the log pointwise predictive density (LPPD), defined as:

$$LPPD=\sum\nolimits_{i=1}^n\log \int p\left({y}_i|\theta \right){p}_{post}\left(\theta \right) d\theta \approx \sum\nolimits_{i=1}^n\log \frac{1}{M}\sum\nolimits_{m=1}^Mp\left({y}_i|{\theta}^{(m)}\right).$$

Just like DIC, there are variants of WAIC which depend on how *p*_*WAIC*_ is defined. Gelman, Hwang, and Vehtari also propose ${p}_{WAIC2}=\sum_{i=1}^n{Var}_{post}\left[\log p\left({y}_i|\theta \right)\right]$ as a penalty term, where *p*_*WAIC*2_ is “the variance of individual terms in the log predictive density summed over the n data points” [48]. Although DIC is a commonly used measure to compare Bayesian models, WAIC has several advantages over DIC, including that it closely approximates Bayesian cross-validation, it uses the entire posterior distribution and it is invariant to parameterisation [49].

Exact cross-validation requires re-fitting the model with different training sets. Approximate leave-one-out cross-validation (LOO-CV) can be computed easily using importance sampling [50]. The Bayesian LOO estimate of out-of-sample predictive fit is

$$LOO=-2{LPPD}_{LOO}=-2\sum_{i=1}^n\log \int p\left({y}_i|\theta \right){p}_{post}\left(\theta |{y}_{-i}\right) d\theta$$

where *p*_*post*_(*θ*| *y*_−*i*_) is the posterior distribution based on the data without the *i*-th data point. Unlike LPPD that uses data point *i* for both the computation of posterior distribution and the prediction, here *LPPD*_*LOO*_ only uses it for prediction, and hence there is no need for a penalty term to correct the potential bias introduced by using data twice [51].

The question regarding the real data is whether the data had better support a true model. To that end, we fit each model using OpenBUGS and compute DIC and LPML for each. We also export the joint posterior distributions from OpenBUGS into R and compute WAIC and LOO with “loo” package [52].

## Numerical study

### Specific models and implementation

In this section, we apply the zero-augmented gamma with random effects and zero-augmented beta-prime with random effects to analyze the multilevel pharmaceutical expenditure dataset previously described, where response (*y*_*ijk*_) is the total pharmaceutical expenditure ($USD) for all drugs prescribed during a 1 year period related to the subject *k* (*k* = 1, …, 29,354) that nested within city *j* (*j* = 1, …, 429) that are nested within province *i* (*i* = 1, …, 31). From now on, the zero-augmented gamma regression model and zero-augmented beta-prime regression model with multilevel random effects, will be called ZAG-RE model and ZABP-RE model, respectively.

**Fig. 2:**
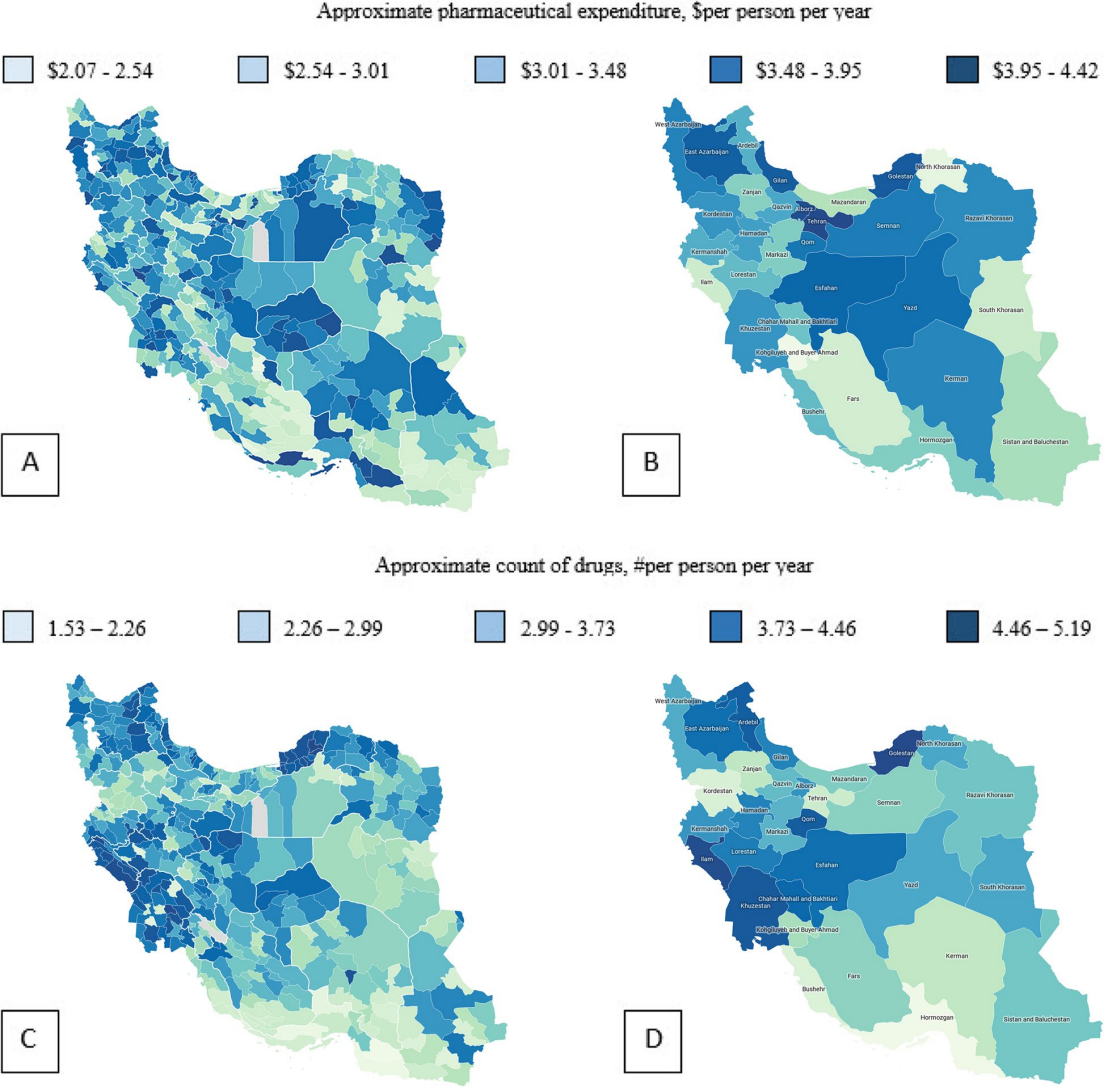
Quintiles of adjusted pharmaceutical expenditure (PE-2018) and counts of drugs by provinces (right) and cities (left) in Iran in 2018. **A**, **B** Variation in adjusted pharmaceutical expenditure. **C**, **D** Variation in adjusted counts of drugs

Figure 2(a-d), shows the quintiles of adjusted pharmaceutical expenditure and counts of drugs by provinces and cities in Iran in 2018. Variation in pharmaceutical expenditure and counts of drugs among clusters (provinces and cities) is well shown. The PE-2018 dataset contains 16.1% of observations with no cost for drugs during the 2018 year. In addition, there is accentuated asymmetry in the empirical distribution of the positive responses, which is confirmed by the sample skewness and sample quartiles (Table 1). These results proposed a skew distribution as a candidate for fitting the pharmaceutical expenditure.

**Table 1:** Percentage of zeros and descriptive statistics of positive expenditure by sex in PE-2018 dataset

	Female (n_1_ = 17,157)	Male (n_2_ = 12,197)	Total (*n* = 29,354)
Number (%) of zeroes	2576 (15.01%)	2147 (17.60%)	4723 (16.10%)
Mean (USD$)	2.8306	2.4397	2.6682
Standard deviation	12.4804	10.8963	11.84928
Coefficient of variation	4.4091	4.4662	4.440926
Skewness	19.128	28.746	22.3550
25%	0.3236	0.270	0.3024
50%	0.9108	0.8640	0.8928
75%	2.2392	2.2374	2.2392

PE-2018 data

Here we model *τ*_*ijk*_ and *μ*_*ijk*_ as follows:

$$logit\left({\tau}_{ijk}\right)={\boldsymbol{X}}_{1 ij k}^{\prime}\times \boldsymbol{\alpha} +{p}_{1i}+{c}_{1 ij}\ \textrm{and}\ \log \left({\mu}_{ijk}\right)={\boldsymbol{X}}_{2 ij k}^{\prime}\times \boldsymbol{\beta} +{p}_{2i}+{c}_{2 ij}$$

where $${\boldsymbol{X}}_{1 ijk}^{\prime }$$ and $${\boldsymbol{X}}_{2 ijk}^{\prime }$$ are the matrices of population effects related to subject *k*, containing an intercept and the following six covariates: Total_*k*_ (total inpatients expenditure ($) per year), ICPD_*k*_ (insurance coverage for prescription drugs per year), NDPP_*k*_ (number of drugs per prescription), NOP_*k*_ (number of prescriptions), Age_*k*_ (in year), and Sex_*k*_ (1 = male, 0 = female); ***α*** and ***β*** are coefficient vectors for the mean and zero-part regression, respectively; *p*_1*i*_ and *p*_2*i*_ be the correlated random effects in the province level with joint density (*p*_1*i*_, *p*_2*i*_) for parts I and II of our model, respectively. Similarly, define *c*_1*ij*_ and *c*_2*ij*_ to be the correlated random effects with joint density (*c*_1*ij*_, *c*_2*ij*_) in the city level.

In the absence of historical data/experiment, our prior choices follow the specifications described in Model specification. Thus, we consider the following independent (weak) priors for the MCMC sampling:

$${a}_r\sim N\left(0,{10}^4\right),r=1,2,3,$$

$${\beta}_q\sim N\left(0,{10}^4\right),q=1,\dots, 7,$$

$${\boldsymbol{\Sigma}}_1^{-1}\sim IW\left(0.01{I}_2,2\right),{\boldsymbol{\Sigma}}_2^{-1}\sim IW\left(0.01{I}_2,2\right)\ \textrm{and} \ \textrm{finally}$$

$$\psi \sim IG\left(0.01,0.01\right).$$

We generate two parallel independent MCMC runs of size 200,000 – each of them with widely dispersed initial values – and discard the first 100,000 iterations (burn-in samples) for later computing of posterior estimates. We consider a lag of size 100 to eliminate potential problems due to autocorrelation and monitor the convergence of the MCMC chains using trace plots and the R statistic [53], which indicates convergence of about 1. To improve convergence, we divide the response (*Y*_*ijk*_) by 100.

### Estimation and model comparison

**Fig. 3:**
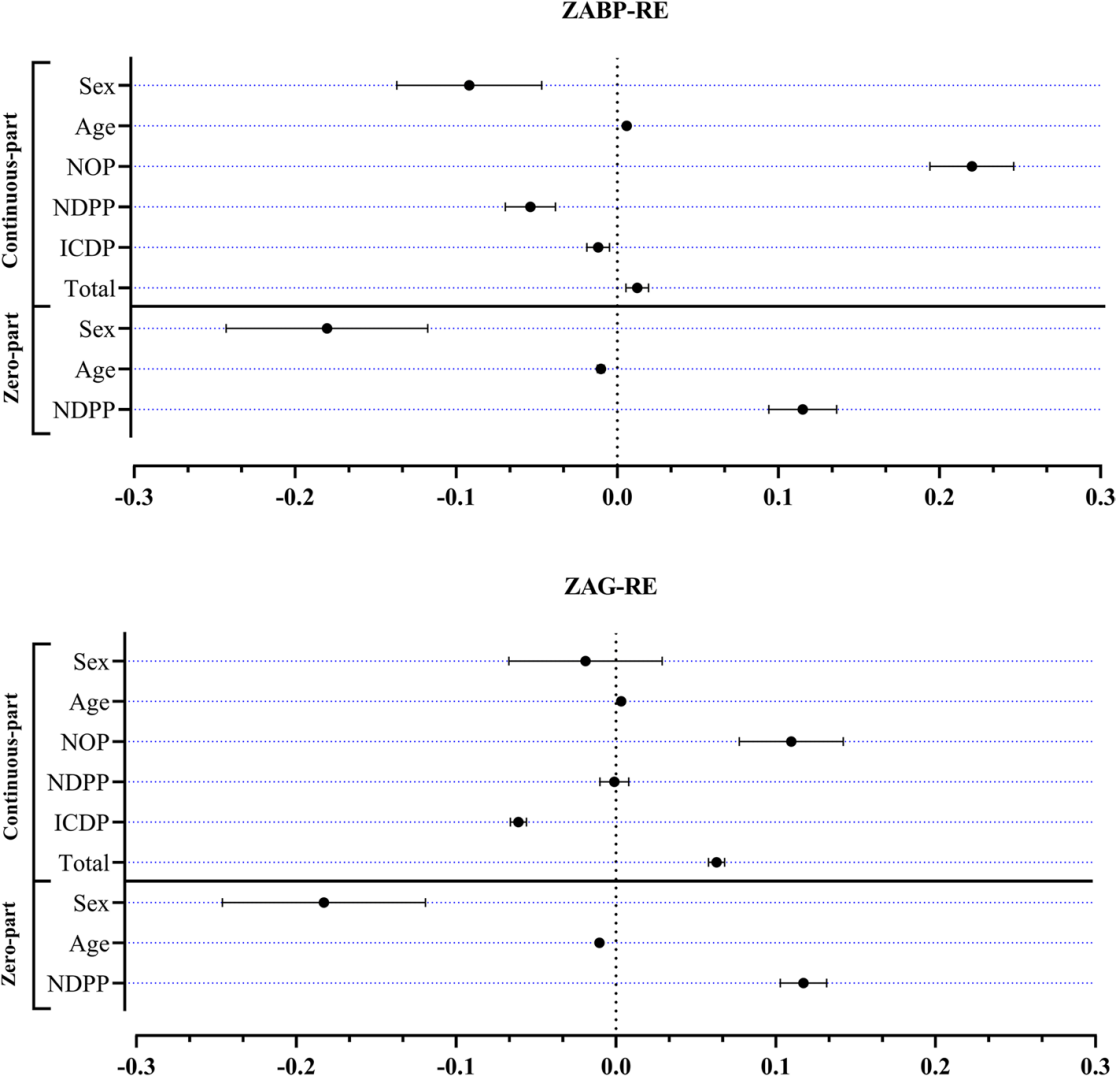
PE-2018 data. Posterior medians and 95% equal-tail credible intervals for parameters associated with fixed effects on the zero-part (Part I) and on the continuous-part (Part II) for the zero-augmented gamma regression and zero-augmented beta-prime regression models

We consider significant those effects whose 95% equal-tail credible intervals (CI) do not include zero (Table 2 and Fig. 3). Except NDPP and sex in continuous part of ZAG-RE, 95% equal-tail CIs show that, all other variables were significant in two parts of models (Fig. 3). Therefore, the final ZAG-RE and ZABP-RE models have, respectively, the following systematic setting:

10 $$\begin{array}{l}{logit}\left(\tau_{ijk}\right)=\alpha_{0}+\alpha_{1}\times{NDPP}_{k}+\alpha_{2}\times{Age}_{k}+\alpha_{3}\times{Sex}_{k}+p_{1i}+c_{1ij}\;\text{and}\\ \log\left(\mu_{ijk}\right)=\beta_{0}+\beta_{1}\times{Total}_{k}+\beta_{2}\times{ICPD}_{k}+\beta_{3}\times{NDPP}_{k}+\beta_{4}\times{NOP}_{k}+\beta_{5}\times{Age}_{k}+\beta_{6}\times{Sex}_{k}+p_{2i}+c_{2ij}.\end{array}$$

**Table 2:** Bayesian selection criteria and posterior estimates of the ZAG-RE and ZABP-RE models fitted to pharmaceutical expenditure (PE-2018) data

	**Criterion**	**ZAG-RE**				**ZABP-RE**			
	DIC_3_	6754.54				6369.19			
	LPML	− 1124.56				− 1124.28			
	WAIC	6769.13				6398.17			
	LOO-CV	6772.95				6403.25			
	Compute time	4195 s				4338 s			
**Model**	**Parameter**	**Posterior features**							
		**Mean**	**SD**	**2.5%**	**97.5%**	**Mean**	**SD**	**2.5%**	**97.5%**
Zero-part	Intercept	1.645	0.051	1.546	1.744	1.234	0.051	1.522	1.714
	NDPP	0.117	0.007	0.103	0.132	0.117	0.003	0.093	0.135
	Age, year	−0.010	0.001	− 0.012	− 0.009	− 0.011	0.001	−0.012	− 0.008
	Male	−0.183	0.032	−0.119	− 0.246	− 0.184	0.024	− 0.116	−0.241
Continuous-part	Intercept	−2.320	0.050	−2.419	− 2.222	−0.988	0.015	−1.069	−0.906
	Total	0.063	0.002	0.058	0.068	0.013	0.00	0.013	0.013
	ICPD	−0.061	0.003	−0.066	− 0.056	−0.012	0.00	−0.013	− 0.12
	NDPP	−0.001	0.005	−0.010	0.008	−0.038	0.003	−0.045	−0.031
	NOP	0.110	0.017	0.077	0.142	0.220	0.013	0.194	0.246
	Age, year	0.003	0.001	0.002	0.005	0.006	0.001	0.004	0.007
	Male	−0.019	0.025	−0.067	0.029	−0.092	0.023	−0.137	− 0.047
	*ψ*	0.905	0.014	0.877	0.933	0.499	0.006	0.487	0.512
Variance component	${\sigma}_{p_1}$	0.144	0.015	0.117	0.176	0.148	0.011	0.112	0.153
	${\sigma}_{p_2}$	0.020	0.011	0.008	0.045	0.019	0.011	0.008	0.041
	${\sigma}_{p_1{p}_2}$	−0.004	0.014	−0.029	0.020	−0.004	0.008	−0.022	0.020
	${\sigma}_{c_1}$	0.718	0.216	0.404	1.250	0.754	0.214	0.397	1.241
	${\sigma}_{c_2}$	13.53	16.51	1.097	56.570	10.16	4.891	1.090	35.525
	${\sigma}_{c_1{c}_2}$	−1.698	1.249	−5.018	0.154	−1.700	0.891	−3.851	0.151

SD, 2.5 and 97.5% represents standard deviation and percentiles from the posterior distributions of parameters, respectively. Computational time in second

*DIC*_*3*_ deviance information criterion, *LPML* log pseudo marginal likelihood, *WAIC* watanabe-akaike information criterion, *LOO-CV* leave-one-out cross-validation, *ZAG-RE* zero-augmented gamma regression, *ZABP-RE* zero-augmented beta-prime regression

The posterior estimates of parameters of model (Eq. 10) shown in Table 2, are quite close in both ZAG-RE and ZABP-RE models while estimates of variance components differ between them. However, 95% equal-tail CI for ${\sigma}_{p_1{p}_2}$ and ${\sigma}_{c_1{c}_2}$ includes zero in both models, indicating no correlation between variances in level 2 and level 3. Posterior standard deviations of variance components are a bit larger under the ZAG-RE model. Also, it is important note that the meaning of parameter *ψ* differs between the ZAG-RE and ZABP-RE models. In ZAG-RE model, *ψ* represents the dispersion parameter, while in ZABP-RE model, it represents the invariance of *Y*_*ijk*_, conditioned on the random effects.

We use DIC_3_, LPML, WAIC, and LOO as the Bayesian model selection criteria and measures of divergence discussed previously to compare the ZAG-RE and ZABP-RE to fit the PE-2018. Except in computational time, The ZABP-RE model performs better according to all other criteria, because it has the smaller DIC_3_, WAIC, and LOO-CV and greater LPML (Table 2). Based on those results, we select the ZABP-RE as our best model.

We also conducted a sensitivity analysis on the prior assumptions for the dispersion parameter (*ψ*) and the fixed effects precision parameter. In particular, we allowed that the dispersion *ψ* ∼ Gamma (k, k) with k ∈ {0.001, 0.1} and the normal precision on the fixed effects to be 0.1, 0.25 and 0.001. We checked the sensitivity in the posterior estimates of ***β*** by changing one parameter at a time and refitting both models. Although slight changes were observed in parameter estimates and model comparison values, the results appeared to be robust and did not change our conclusions regarding the best model, inference, and sign of the fixed-effects.

From these findings, we further report the results in detail only for the best ZABP-RE model in the following Section.

### Results for the ZABP-RE model

We use the ZABP-RE model to interpret parameters effects on the mean of positive expenditure (*μ*_*ijk*_) and the probability of non-cost (*τ*_*ijk*_) by considering individual effects as zero. To measure effects directly on *μ*_*ijk*_ and *τ*_*ijk*_, we take the anti-logarithm of log(*μ*_*ijk*_) and logit(*τ*_*ijk*_) in Eq. (10), obtaining

11 $${\displaystyle \begin{array}{c}{\mu}_{ijk}=\exp \left({\beta}_0+{\beta}_1{Total}_k+{\beta}_2{ICPD}_k+{\beta}_3{NDPP}_k+{\beta}_4{NOP}_k+{\beta}_5{Age}_k+{\beta}_6{Sex}_k\right)\\ {}\textrm{and}\\ {}{\tau}_{ijk}=\frac{\exp \left({\alpha}_0+{\alpha}_1\times {NDPP}_k+{\alpha}_2\times {Age}_k+{\alpha}_3\times {Sex}_k\ \right)}{1+\exp \left({\alpha}_0+{\alpha}_1\times {NDPP}_k+{\alpha}_2\times {Age}_k+{\alpha}_3\times {Sex}_k\right)}\end{array}}$$

We use the posterior means in Table 2 as estimates of the parameters. From Eq. (11), parameter *β*_*i*_ represents the rate of change in the logarithm of the mean of the positive expenditure as each of total, ICPD, NDPP, NOP, and age increases one unit. Therefore, increasing NOP variable of subject *k* in the original scale by one, the log (*μ*_*ijk*_) increases by 0.22, where exp(0.22) = 1.25 is the increasing value of response variable in its original scale. Parameters *α*_0_, *α*_1_, *α*_2_ and *α*_3_ contribute to the calculation of *τ*_*ijk*_ in Eq. (11). Here, *α*_0_ represents the effect of being a female respondent with age and NDPP set to their respective mean. Setting NDPP and age variables to zero – which implies they are set to their respective mean in the original scale – the probability of no consumption is 1 − *τ*_*ijk*_ = 1 −  *exp* (1.645)/(1 + exp(1.645) ) = 0.16 if subject *k* is female and 1 − *τ*_*ijk*_ = 1 −  *exp* (1.645 − 0.183)/(1 + exp(1.645 − 0.183) ) = 0.19 if subject *k* is male. Overall, females tend to declare a larger expenditure since the estimate of *α*_3_ is negative. Parameters *α*_1_ (0.117) and *α*_2_ (−0.010) represent the effect of NDPP and age variables in logit (*τ*_*ijk*_). In particular, as NDPP variable increase by one unit, with every additional pharmaceutical item, the odds of having a positive expenditure increase by 12.41%. In addition, with each year growing in age, the Odds of having a positive cost decreases by 1%.

**Fig. 4:**
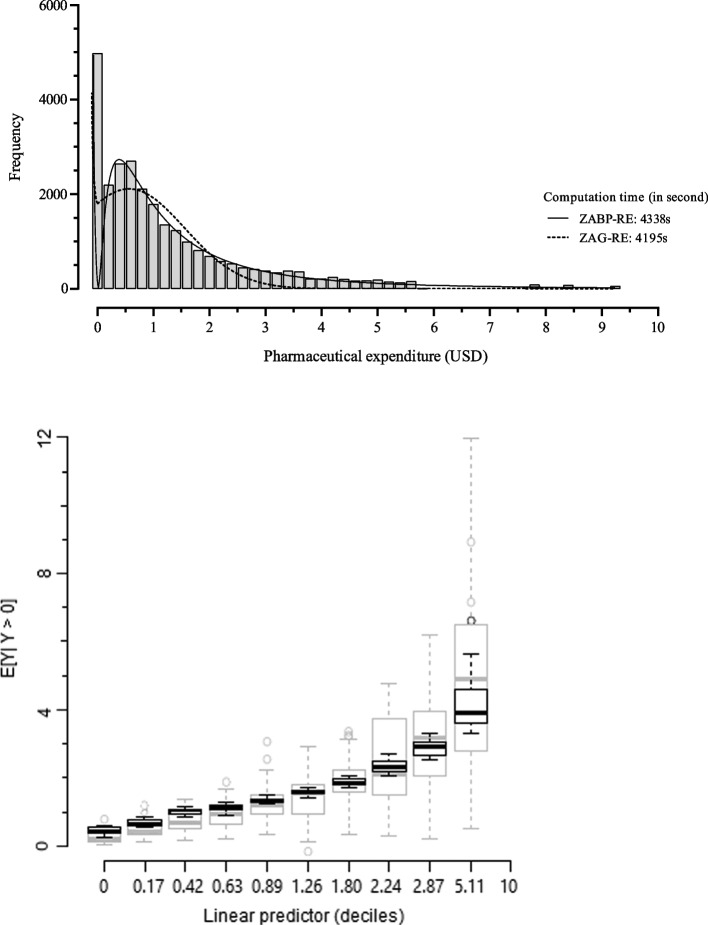
PE-2018 data. (Above panel) predictive density histogram from pharmaceutical expenditure placed with posterior predictive densities generated using ZABP-RE regression models. (Below panel) adequacy of log link function linear predictor: conditional predictive posterior nonzero mean represented by black boxplots and nonzero observed values by gray boxplot

In order to evaluate the predictive performance of our best model, we generate 2000 replicates of 𝐘, say, ***Y***^***∗***^ = (*Y*^(1)^, …, *Y*^(2000)^ )^*T*^. The *ijk*-th element of the *l*-th replicate ${Y}_{ijk}^{(l)}$ is generated through the ZABP-RE(${\mu}_{ijk}^{(l)},{\phi}^{(l)},{\tau}_{ijk}^{(l)}$) model, where ${\mu}_{ij k}^{(l)}={{\log}}^{-1}\left(\sum_{\theta =1}^q{\boldsymbol{\beta}}_{\theta}^{(l)}{x}_{\theta_i}+{p}_i^{(l)}+{c}_{ij}^{(l)}\right)$ and ${\tau}_{ij k}^{(l)}={logit}^{-1}\left(\sum_{\gamma =1}^r{\boldsymbol{\alpha}}_{\gamma}^{(l)}{x}_{\gamma_i}+{p}_i^{(l)}+{c}_{ij}^{(l)}\right)$. The values of ${\boldsymbol{\alpha}}^{(l)}=\left({\alpha}_0^{(l)},\dots, {\alpha}_3^{(l)}\right)$, ${\boldsymbol{\beta}}^{(l)}=\left({\beta}_0^{(l)},\dots, {\beta}_6^{(l)}\right)$ and *ϕ*^(*l*)^ are post burn-in samples of size 2000 from the posterior distribution of all parameters. Figure 4 (above panel) presents the histogram of PE-2018 placed with the plot of the ZABP-RE and ZAG-RE predictive posterior density. In this figure, it is also quite clear that with a slightly more computational time, the ZABP-RE model provides an adequate fit to the PE-2018 data.

Finally, to evaluate the adequacy of the log-link function used to model the conditional nonzero mean *μ*, we follow the suggestion given in [54] as depicted in Fig. 4 (below panel). We divide the values of the linear predictor *μ*_*ijk*_ into 10 intervals, with each interval containing a similar number of observations. Then, for each group, we build a boxplot of the posterior predictive mean (black boxplot) and a boxplot of the nonzero observed values (gray boxplot). In Fig. 4, we observe no evidence of link misspecification for the nonzero mean *μ*_*ijk*_, because the shapes of the fitted and observed trends are similar.

### Simulations

In this section, we propose a simulation study to illustrate the performance of the proposed method. Our goals for the simulation study were: 1) to investigate the behaviour of Bayesian estimates based on the empirical mean squared error (MSE), relative bias and percentage of times that the 95% credible intervals (CI) contains the true parameter value and 2) to investigate if the DIC_3_ and LPML Bayesian criteria properly select the best model. We conduct the simulation study considering 100 datasets generated from ZAG-RE and ZABP-RE models, considering different sample sizes n, say *n* = 50, 100, 150 and 200. For each dataset of size n, we model the location parameter *μ*_*ijk*_ through log(*μ*_*ijk*_) = *β*_0_ + *β*_1_*x*_*ijk*_ + *p*_*i*_ + *c*_*ij*_, with ${p}_i\sim N\left(0,{\sigma}_p^2\right)$ and ${c}_{ij}\sim N\left(0,{\sigma}_c^2\right)$, *i* = 1, …, n, *j* = 1, …, *n*_*i*_, *k* = 1, …, *m*_*ij*_. To keep the simulation simple and fast, *τ* is considered constant across observations. We generate independent explanatory variables *X*_*ijk*_ from a Bernoulli distribution with a parameter equal to 0.8 and set *β*_0_ = 2, *β*_1_ = 1.5, *τ* = 0.2, ${\sigma}_p^2=1.8$, ${\sigma}_c^2=2.3$, *ψ* = 1.0 for the ZAG-RE model and *β*_0_ = 2, *β*_1_ = 1.5, *τ* = 0.2, ${\sigma}_p^2=1.8$, ${\sigma}_c^2=2.3$, *ψ* = 0.1 for the ZABP-RE model. We consider the following independent non-informative priors *β*_*k*_~*N*(0,100), *ψ*~*Gamma*(0.01, 0.01), ${\sigma}_p^2\sim IGamma\left(0.01,0.01\right)$, ${\sigma}_c^2\sim IGamma\left(0.01,0.01\right)$ and *τ*~*U*(0, 1).

For each dataset of size n, we calculate Bayesian estimates with 500 points from the posterior distribution. These points are based on two parallel independent MCMC runs of size 100,000 each, discarding the first 50,000 points to eliminate the effect of the initial values. To avoid correlation among observations, we consider a thinning of size 100, obtaining 500 points from the posterior distribution.

**Fig. 5:**
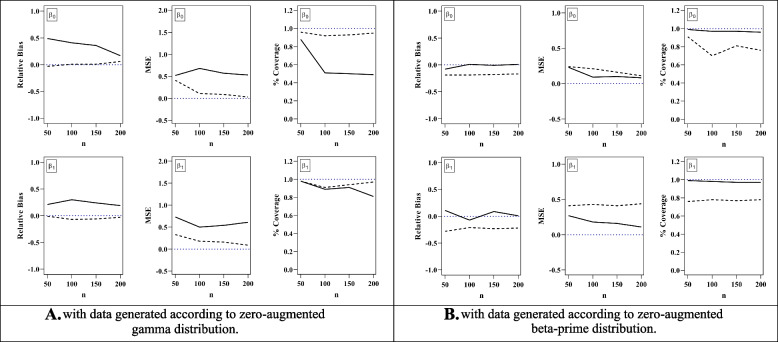
Simulation study. Relative bias, mean squared error, and coverage probability for *β*_0_ and *β*_1_ using the zero-augmented gamma (⋯) regression and zero-augmented beta-prim (−) regression models

To study the frequentist properties of Bayesian estimates, we calculate the relative bias, the MSE and the 95% coverage probability (CP). Let 𝜽 = { ***α***, ***β***, *ψ*, 𝜎^2^} be the true vector of parameters and 𝜃_s_ an element of 𝜽. Let ${\hat{\theta}}_s$ be the posterior mean of 500 points from the posterior distribution of 𝜃_s_ based on dataset *i* of size *n*, *i* = 1, …, 100, *n* = 50, 100, 150, 200. The relative bias, the MSE and the 95% CP for ${\hat{\theta}}_s$ are defined as follows:

$$\mathrm{Relative}\;\mathrm{bias}\;\left({\widehat\theta}_s\right)=\frac1{100}\sum_{i=1}^{100}\left(\frac{{\widehat\theta}_{is}-\theta_s}{\theta_s}\right)$$

$$\mathrm{MSE}\;\left({\widehat\theta}_s\right)=\frac1{100}\sum_{i=1}^{100}\left({\widehat\theta}_{is}-\theta_s\right)^2$$

$$\mathrm{CP}\left({\widehat\theta}_s\right)=\frac1{100}\sum_{i=1}^{100}I\left(\theta_s\in\left[{\widehat\theta}_{is,LCL},{\widehat\theta}_{is,UCL}\right]\right)$$

where *I* is the indicator function such that *θ*_*s*_ lies in the interval $\left[{\hat{\theta}}_{is, LCL},{\hat{\theta}}_{is, UCL}\right]$, with ${\hat{\theta}}_{is, LCL}$ and ${\hat{\theta}}_{is, UCL}$ as the estimated lower and upper 95% CIs, respectively. Fig. 5A and B present a visual comparison of the parameters *β*_0_ and *β*_1_ under ZAG-RE and ZABP-RE generated data for varying sample sizes, where the dotted and black lines represent the ZAG-RE and the ZABP-RE fitted model, respectively.

As expected, these figures reveal that if we use a true (ZAG-RE and ZABP-RE) model to fit zero augmented-skew data, relative bias and MSE, for parameters *β*_0_ and *β*_1_, tend to decrease as sample size increases indicating that the Bayesian estimates possess good consistency properties. In addition, both CP of *β*_0_ and *β*_1_ tend to be around 95% as the sample size increases when the true model is considered. However, when we do not fit the data by their respective true model (model misspecification), the relative bias and the MSE tend to be smaller in ZABP-RE model. Moreover, as expected in both cases we can see that the performance of the CP gets worse when a misspecified model is considered. For the sake of completeness, the MSE, relative bias and CP for all the parameters (*β*_0_, *β*_1_, *τ*, *σ*^2^) are presented in Table B1 (Additional file 1: Appendix B). It can be seen from this table that the Bayesian estimates of the mixture proportion *τ* are highly robust to model misspecification, and this behaviour is independent of the sample size. The dispersion parameters (*σ*^2^ and *ψ*) in both models are not comparable, because they are in different scale. In all results, relative bias of ${\sigma}_c^2$ are greater than ${\sigma}_p^2$ and it shows that the dispersion of responses is more at the level-2 than the level-3. In the comparison of the two models, the differences in MSE values of both ${\sigma}_p^2$ and ${\sigma}_c^2$ are absolutely more in misspecified model. The results of coverage probability of sigmas show that the coverage level in both models are acceptable, however, in true models, the values of the CP are slightly larger than the misspecified model.

Summary of model performance in simulations are presented in Table 3. Table 3 presents the averages of the Bayesian model comparison criteria. We calculate the LPML, DIC_3_, WAIC, LOO, and convergence rate using 100 samples of size *n* = 100 each. All criteria favoured the true (simulated) model.

**Table 3:** Summary of model performance in the simulation study

		Fitted Model	
True Model	Criterion	ZAG-RE	ZABP-RE
ZAG-RE	LPML	− 4536.87	− 6413.12
	DIC_3_	5.12 × 10^6^	5.88 × 10^7^
	WAIC	5.14 × 10^6^	5.89 × 10^7^
	LOO-CV	5.18 × 10^6^	5.93 × 10^7^
	CR%	98%	98%
ZABP-RE	LPML	− 9659.82	− 8230.09
	DIC_3_	8.45× 10^8^	5.83× 10^8^
	WAIC	8.96 × 10^8^	5.89 × 10^8^
	LOO-CV	9.11 × 10^8^	6.13 × 10^8^
	CR%	96%	99%

Simulation study

*LPML* log pseudo marginal likelihood, *DIC*_*3*_ deviance information criterion, *WAIC* watanabe-akaike information criterion, *LOO-CV* leave-one-out cross-validation, *CR* convergence rate, *ZAG-RE* zero-augmented gamma with random effects, *ZABP-RE* zero-augmented beta-prime with random effects

## Discussion

In this article, we proposed a Bayesian mixture model with random effects for modelling semi-continuous data augmented by zeros. We suggest the Gamma and BP distributions in continuous part of the models. A simulation study and real data analysis are conducted to compare the ZAG-RE and ZABP-RE on the multi-level semi-continuous data and results demonstrated that the ZABP-RE performs better on the zero-augmented multilevel semi-continuous data.

Our flexible class contains the zero-augmented versions of the two parametric exponential family of distributions, such as Gamma, beta-prime, inverse Gaussian, Weibull, log-normal, and Tweedie. Our model is able to simultaneously accommodate zeros and positive outcomes, right-skewness, within subject correlation because of nested measurements and between-subject heterogeneity. One of the differentials of this study was the inclusion of random effects in the analysis of factors related to semi-continuous data using the beta-prime distribution that were not considered in before studies and statistical packages [10, 20–22], and this is our major contribution.

One of the advantages of the Bayesian approach compared to the classical approach is the estimates in the part I. Where, the maximum likelihood estimator of a probability of non-zero value, when zero response is observed, does not perform well on the boundary of the parameter space [37]. For a simple BP model, the Maximum Likelihood estimation is available using GAMLSS. However, the MLE results in our data did not reach convergence for some parameters by adding random effects. In this research, using Bayesian statistics with Gibbs and Metropolis-Hasting sampling, this problem is avoided. In the future, it would be interesting to continue the study of various different MCMC methods and hopefully apply them to health cost data.

Simulation studies reveal good consistency properties of the Bayesian estimates as well as high performance of the model selection techniques to pick the appropriately fitted model. We also apply our model to a dataset from yearly pharmaceutical expenditure data conducted in 429 cities in Iran (PE-2018) to illustrate how the procedures can be used to evaluate model assumptions and obtain unbiased parameter estimates. Although our modelling is primarily motivated from the PE-2018, it can be easily applied to other datasets and distributions, because the models considered in this article have been fitted using standard available software packages, like *R* and OpenBUGS (code available in Additional file 1: Appendix A). This makes our approach easily accessible to practitioners of many fields of research.

Although the zero-augmented positive model considered here has shown great flexibility to deal with zero-augmented clustered data, its robustness can be seriously affected by the presence of heavy tails in the random effects, obscuring important features among individual variation. Liu et al. [13] and Bandyopadhyay et al. [55] proposed a remedy to accommodate skewness in the random effect simultaneously, using skew-normal/independent distributions. We suppose that our method can be used under the zero-augmented positive model and should yield satisfactory results at the expense of additional complexity in implementation. Another useful extension of the proposed model involves the possibility of heteroscedasticity of *ψ* by allowing the dependence of g(*ψ*) on covariates, with g(.) being an appropriate link function, as proposed in [56]. An in-depth investigation of such extensions is beyond the scope of the present research article, but it is an interesting topic for further research.

## Supplementary Information

**Additional file 1:** **Appendix A.1.** OpenBUGS code. **Appendix B.** Results of the simulations. **Table B1.** Relative bias, MSE and CP for parameter estimates with different sample sizes for the ZAG-RE and ZABP-RE models.

## Data Availability

The data that support the findings of this study are available from the corresponding author reasonable request.

## Abbreviations

PE: Pharmaceutical expenditure
BP: Beta-prime
LMMs: Linear mixed models
DDD: Daily drinks data
GLMMs: Generalized linear mixed models
MCMC: Markov Chain Monte Carlo
M-H: Metropolis-Hastings
LPML: Log pseudo marginal likelihood
DIC: Deviance Information Criterion
CPO: Conditional predictive ordinate
WAIC: Watanabe-akaike information criterion
LOO-CV: Leave-one-out cross-validation
CR: Convergence rate
ZAG-RE: Zero-augmented gamma regression model with random effects
ZABP-RE: Zero-augmented beta-prime regression model with random effects
NCHIR: National Center for Health Insurance Research
