# Supplementary material for: Zero-augmented beta-prime model for multilevel semi-continuous data: a Bayesian inference
Source: BMC Med Res Methodol. 2022 Nov 2;22:283. doi: 10.1186/s12874-022-01736-0 (PMC9628168; doi:10.1186/s12874-022-01736-0)
Supplement: Supplementary file 1 — Additional file 1: Appendix A.1. OpenBUGS code. Appendix B. Results of the simulations. Table B1. Relative bias, MSE and CP for parameter estimates with different sample sizes for the ZAG-RE and ZABP-RE models. [file 12874_2022_1736_MOESM1_ESM.docx]

# Appendix A.1: OpenBUGS code

model {

#Random effects

med[1]<-0

med[2]<-0

for (j in 1:429) { u[j,1:2]~dmnorm(med[1:2], Omega[,]) }

for (i in 1:31) { v[i,1:2]~dmnorm(med[1:2], Sai[,]) }

# ZAG-RE model (uncommented model) and ZABP model (commented model)

c<-1000

for(k in 1:29354)

{

zeros[k]<-0

zeros[k]~dpois(zeros.means[k])

zeros.means[k]<- -loglilkAugmented[k]+ c

#The posterior predictive ordinate (PPO)

ppo.term[k] <- exp(-mu[k] + y[k]*log(mu[k]) - logfact(y[k]))

# The conditional predictive ordinate (CPO)

icpo.term[k] <- 1/ppo.term[k]

log.icpo[k]<- log(icpo.term[k])

#pdf of Gamma distribution

pdf[k]<-exp(-loggam(phi)+phi*(log(phi)-log(mu[k]))+(phi-1)*log(y[k])-#(phi*y[k])/mu[k])

#Beta-prime distribution

pdf[k]<-exp((mu[k]*(phi+1)-1)*log(y[k])-(mu[k]*(phi+1)+phi+2)*log(1+y[k])-((loggam(mu[k]*(phi+1)+(phi+2)))-loggam(mu[k]*(phi+1))-loggam(phi+2)))

e[k]<-equals(y[k],0)

loglilkAugmented[k]<-log(e[k]*p[k]+(1-e[k])*pdf[k]*(1-p[k]))

logit(p[k])<-

alfa[1]+alfa[2]*Drug[k]+alfa[3]*Age[k]+alfa[4]*Sex[k]+u[IDC[k],1]+v[IDP[k],1]

log(mu[k])<-

beta[1]+beta[2]*Total[k]+beta[3]*ICPD[k]+beta[4]*NDPP[k]+beta[5]*NOP[k]+beta[6]*Age[k]+beta[7]*Sex[k]+u[IDC[k],1]+v[IDP[k],1]

}

# Prior distributions of the parameters

for (i in 1:4) { alfa[i]~dnorm(0, 0.0001) }

for (i in 1:7) { beta[i]~dnorm(0,0.0001) }

phi~dgamma(0.01, 0.01)

sigma2 <-1/phi

#The log-pseudo marginal likelihood (LPML)

LPML<- sum(log.icpo[1:29353])/29354

# B: Results of the simulations.

| **Table B1.** Relative bias, MSE and CP for parameter estimates with different sample sizes for the ZAG-RE and ZABP-RE models. | | | | | | | | | |
| --- | --- | --- | --- | --- | --- | --- | --- | --- | --- |
|  |  | Fitted model | | | | | | | |
|  |  | ZAG-RE | | | | ZABP-RE | | | |
| True model | Parameter | 50 | 100 | 150 | 200 | 50 | 100 | 150 | 200 |
|  |  | **Relative bias** | | | | | | | |
| ZAG-RE | $\beta_{0}$ | -0.03 | 0.01 | 0.01 | 0.06 | 0.49 | 0.41 | 0.36 | 0.17 |
|  | $\beta_{1}$ | -0.01 | -0.07 | -0.06 | -0.03 | 0.21 | 0.30 | 0.24 | 0.19 |
|  | $\psi$ | 0.03 | 0.02 | 0.01 | 0.01 | 0.14 | 0.05 | 0.08 | 0.06 |
|  | $\sigma_{p}^{2}$ | 2.50 | 2.47 | 2.44 | 2.39 | 3.14 | 3.02 | 3.02 | 2.14 |
|  | $\sigma_{c}^{2}$ | 4.78 | 4.46 | 4.65 | 4.34 | 7.01 | 6.54 | 6.50 | 5.98 |
| ZABP-RE | $\beta_{0}$ | -0.19 | -0.19 | -0.18 | -.017 | -0.08 | 0.01 | -0.01 | 0.01 |
|  | $\beta_{1}$ | -0.28 | -0.21 | -0.23 | -0.22 | 0.11 | -0.07 | 0.09 | 0.01 |
|  | $\psi$ | 0.17 | 0.11 | 0.08 | 0.06 | 0.04 | 0.03 | 0.01 | 0.01 |
|  | $\sigma_{p}^{2}$ | 3.04 | 3.02 | 3.02 | 2.84 | 2.52 | 2.89 | 2.38 | 2.39 |
|  | $\sigma_{c}^{2}$ | 5.91 | 6.14 | 5.50 | 5.15 | 3.22 | 4.01 | 3.18 | 3.07 |
|  |  | **MSE** | | | | | | | |
| ZAG-RE | $\beta_{0}$ | 0.41 | 0.11 | 0.09 | 0.03 | 0.52 | 0.68 | 0.57 | 0.53 |
|  | $\beta_{1}$ | 0.33 | 0.18 | 0.16 | 0.09 | 0.73 | 0.50 | 0.54 | 0.61 |
|  | $\psi$ | 0.04 | 0.02 | 0.01 | 0.01 | 0.11 | 0.08 | 0.8 | 0.04 |
|  | $\sigma_{p}^{2}$ | 1.61 | 1.60 | 1.57 | 1.55 | 20.32 | 19.12 | 17.89 | 17.42 |
|  | $\sigma_{c}^{2}$ | 2.23 | 2.09 | 1.87 | 1.75 | 17.54 | 12.69 | 13.04 | 11.54 |
| ZABP-RE | $\beta_{0}$ | 0.24 | 0.21 | 0.16 | 0.11 | 0.23 | 0.09 | 0.10 | 0.08 |
|  | $\beta_{1}$ | 0.41 | 0.43 | 0.41 | 0.44 | 0.27 | 0.18 | 0.16 | 0.13 |
|  | $\psi$ | 0.07 | 0.08 | 0.07 | 0.06 | 0.05 | 0.03 | 0.01 | 0.02 |
|  | $\sigma_{p}^{2}$ | 15.21 | 12.39 | 11.98 | 11.53 | 1.89 | 1.63 | 1.57 | 1.54 |
|  | $\sigma_{c}^{2}$ | 12.54 | 8.98 | 8.46 | 7.99 | 2.25 | 2.14 | 1.71 | 1.50 |
|  |  | **%CP** | | | | | | | |
| ZAG-RE | $\beta_{0}$ | 0.98 | 0.97 | 0.98 | 0.99 | 0.88 | 0.51 | 0.50 | 0.49 |
|  | $\beta_{1}$ | 0.98 | 0.91 | 0.94 | 0.97 | 0.98 | 0.89 | 0.91 | 0.81 |
|  | $\psi$ | 0.94 | 0.95 | 0.94 | 0.93 | 0.90 | 0.91 | 0.90 | 0.92 |
|  | $\sigma_{p}^{2}$ | 0.89 | 0.87 | 0.87 | 0.89 | 0.75 | 0.79 | 0.81 | 0.85 |
|  | $\sigma_{c}^{2}$ | 0.85 | 0.88 | 0.91 | 0.92 | 0.88 | 0.89 | 0.87 | 0.89 |
| ZABP-RE | $\beta_{0}$ | 0.91 | 0.70 | 0.81 | 0.76 | 0.99 | 0.97 | 0.97 | 0.96 |
|  | $\beta_{1}$ | 0.76 | 0.78 | 0.77 | 0.78 | 0.99 | 0.98 | 0.97 | 0.97 |
|  | $\psi$ | 0.85 | 0.84 | 0.86 | 0.90 | 0.94 | 0.93 | 0.93 | 0.92 |
|  | $\sigma_{p}^{2}$ | 0.91 | 0.91 | 0.92 | 0.91 | 0.94 | 0.94 | 0.96 | 0.95 |
|  | $\sigma_{c}^{2}$ | 0.94 | 0.95 | 0.93 | 0.92 | 0.95 | 0.97 | 0.96 | 0.97 |
| CP: coverage probability; MSE: mean squared error. ZAG-RE: zero-augmented gamma regression; ZABP-RE: zero-augmented beta-prime regression. | | | | | | | | | |
